# Quality Improvement of a Hip Injection Service

**DOI:** 10.7759/cureus.32063

**Published:** 2022-11-30

**Authors:** Haris Duvnjak, Mohannad B Ammori

**Affiliations:** 1 Otolaryngology, Wrexham Maelor Hospital, Wrexham, GBR; 2 Trauma and Orthopedics, Wythenshawe Hospital, Manchester, GBR

**Keywords:** hip, quality improvement research, time-to-treatment, intra-articular injection, osteoarthritis (oa)

## Abstract

Background

Osteoarthritis is a substantive burden on the population and National Health Service (NHS) in the United Kingdom. A recent systematic review suggests that intra-articular steroid injections are an efficacious conservative treatment modality. The NHS Constitution states that “patients should wait no longer than 18 weeks from GP referral to treatment.” An NHS hospital trust failed to meet this standard in a quarter of patients waiting for an intra-articular steroid injection of the hip. Strategies were considered to improve the time from referral to treatment (RTT). The aim of this quality improvement project was to improve the efficiency and capacity of the injection list.

Materials and methods

Patients who underwent an injection on a list between January and April 2019 were identified. Data were retrospectively collected and included the sites of injection and waiting times. Proformas for documentation and discharge summaries were introduced. Time taken for each appointment pre- and post-intervention were compared with the aim to increase the number of patients per list.

Results

Thirty-six (26%) of 138 patients experienced delays beyond 18 weeks from RTT. The mean (standard deviation [SD]) number of weeks waiting for an injection from the time of referral was 14 (6). The mean (SD) time for each appointment was 12 minutes 36 seconds (7 minutes 36 seconds) pre-intervention and 8 minutes 36 seconds (3 minutes 12 seconds) post-intervention.

Discussion and conclusion

Our standardized proformas led to an improvement in efficiency by reducing the time taken for documentation and capacity by subsequently increasing the number of patients per list.

## Introduction

Background

Hip osteoarthritis (OA) is a degenerative disease that affects the hip and is generally defined by a loss of joint space and damage to the articular cartilage. It can be classified into mild, moderate, and severe according to radiological criteria. In moderate to severe cases, there can be an effusion or other elements of joint capsule inflammation. The presence of pathological features and severity varies between patients, but nearly all complain of pain emanating from the joint, usually groin pain that worsens upon physical activity. It is the second most common form of peripheral joint osteoarthritis after knee osteoarthritis. Hip OA is more common in increasing age, being greatest in those >75 years, and twice more common in females than males. It is thought that the cause is multifactorial, with a combination of age, sex, genetics, dysplasia, joint laxity, and high BMI implicated [1].

Hip OA can have a huge detrimental impact on the quality of life of patients from activity-related pain and reduced mobility. Patients report difficulties in walking and sleeping, the need for walking aids, giving up active hobbies, giving up driving, and poor self-esteem. It is associated with morbidity, frailty, and higher rates of mental health problems. It also has a huge impact on wider society with 10.9% of people (2.46 million people) being affected in the United Kingdom. Of these people, 86.2% (2.12 million) have sought treatment. In central Manchester where this project was done, it is slightly more prevalent at 11.4% of the general population [2].

At present, there is no therapy that slows the disease progression of osteoarthritis. The main goal of management is to improve the quality of life by multimodal means, with non-pharmacological strategies focusing on modifiable risk factors as well as maintaining function and pharmacological strategies focusing on alleviating pain. Early osteoarthritis is usually managed by regular exercise, steady weight maintenance, and pain management. Surgical management consists of total hip replacement and is usually reserved for later osteoarthritis where patients have poorly responded to the mentioned non-surgical options [3].

Pharmacological management of hip osteoarthritis: guidance and evidence

With regard to pain management, the National Institute for Health and Care Excellence (NICE, 2022) recommends a stepwise approach to analgesia with paracetamol as needed as a starting point. Non-steroidal anti-inflammatories (NSAIDs) such as ibuprofen and naproxen can then be used in place of or in addition to paracetamol [4]. According to a Cochrane review, paracetamol is less effective than NSAIDs in managing hip pain in osteoarthritis, and NSAIDs are more effective in managing hip pain secondary to moderate to severe osteoarthritis. It is thought to be due to the alleviation of synovitis present in moderate to severe OA [5]. However, as NSAIDs are associated with adverse renal, hepatic, and gastrointestinal effects, only shorter courses with a low-dose proton pump inhibitor are recommended.

NICE recommends considering adding an opioid if this fails to manage pain, ideally starting with a low dose of a weaker opioid such as tramadol allowing room to increase doses. A Cochrane review showed that there is a small benefit in pain management with the addition of tramadol. However, substantially more people stop taking tramadol due to adverse effects than paracetamol [6]. A different Cochrane review suggests that while non-tramadol opioids offer a small mean benefit, there is a significant increase in the risk of adverse effects [7]. Osteoarthritis Research Society International (OARSI) recommends stronger opioids in only exceptional circumstances and advises to consider surgical treatment in these cases.

Hip intra-articular steroid injections (IASIs) are considered an adjunct when there is poor response to oral analgesia in moderate to severe osteoarthritis according to NICE (2008) and OARSI. Hip IASIs involve an injection of a steroid with or without a local anesthetic that is performed under either fluoroscopic, x-ray, or ultrasound guidance to visualize the joint. It is usually given three monthly, up to four times a year. It may be that those with an inflammatory element (synovitis or effusion) and chondral injury benefit most, but there is limited data to prove this [8].

IASIs have been used for more than 70 years. Despite this, there have been few RCTs to look at their efficacy. We searched PubMed with “intra-articular,” “hip,” and “systematic review” as keywords and selected the most recent systematic review, conducted by Lynch et al. in 2016. It was chosen as our main source as it analyzed all available randomized control trials (RCT) comparing hip IASIs under imaging guidance. The review found that they are well tolerated with little systemic effects and concluded that they may have a useful role in short-term pain management [9-11]. The most common adverse effect is a flare of pain and swelling around the injection site with serious adverse effects being rare, with only one participant being affected in the review. The potential benefit of the IASIs is a rapid onset of clinically significant pain reduction, with efficacy best at one-week post-injection and transient improvement in function lasting up to eight weeks. However, the authors of the review encountered limitations with the literature, which led to the conclusion that the overall quality of evidence was poor. Dose-dependent efficacy could not be assessed as there were variations between trials on the chosen steroid and dosages, and these have not been compared in an RCT with the same standardized outcome measures.

Furthermore, the population sizes of the RCTs reviewed were small, with only five trials consisting of a total of 346 participants that lasted only short durations [12,13]. Also, not all studies used a saline placebo [14]. This meant that the authors found it difficult to accurately gauge treatment effect size and had to acknowledge the potential of interpreter bias. It must also be said that most patients selected for the RCTs had severe hip osteoarthritis and that any conclusions made on the efficacy of hip injections are not reflective of all degrees of severity. The authors conclude that further larger studies should be conducted to establish dose and time-dependent efficacy and safety profile.

It also has a diagnostic role; if the patient reports improvement in symptoms, it suggests the presence of hip OA. McCabe et al. (2016) demonstrated that nonresponse to injection is a strong negative predictor of hip arthroplasty.

Quality improvement

The NHS Constitution in England states that patients "have the right to access certain services commissioned by NHS bodies within maximum waiting times, or for the NHS to take all reasonable steps to offer a range of suitable alternative providers if this is not possible." The NHS Constitution states that patients should wait no longer than 18 weeks from GP referral to treatment (RTT) [15]. The NHS operation standard for non-admission procedures is 95%. A previous departmental audit in 2019 in a Manchester NHS tertiary center found that it failed to meet this standard in a quarter of patients waiting for an intra-articular steroid injection of the hip. The aim of this quality improvement project was to improve the efficiency and capacity of this injection list.

This study was presented as a poster titled "Improving a Hip Injection Service With Simple Changes" at the International Virtual Medical Conference on May 2020.

## Materials and methods

Patients who underwent an IASI on a hip injection list were identified from the injection lists over a period of four months in 2019 (n = 143). Data were retrospectively collected from the picture archiving and communication system (PACS) and the electronic patient record (EPR) and included the sites of injection (unilateral versus bilateral), the date and time of injection, and the time from RTT. The date of referral was calculated by deducting 18 weeks from the breach date. The quality standard used was 95% as previously mentioned.

IASIs of the hip were performed on the first three Tuesday afternoons of the month in the fluoroscopy suite. Skin preparation was undertaken using povidone-iodine. Iohexol contrast was injected intra-articularly under fluoroscopic guidance to ensure the correct anatomical location before injecting the preparation. The chosen preparation was 80 mg of Depo-Medrone and 4 ml of 0.5% Bupivacaine. The documentation was handwritten in the patients’ notes, and standardized empty transferable non-admissions procedure proformas were used to document the discharge summaries.

**Figure 1:**
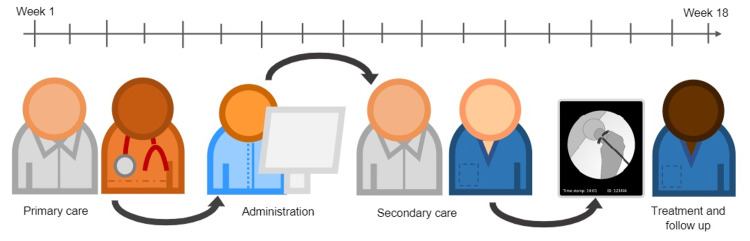
Timeline of the patient journey from referral to treatment

Figure 1 illustrates the timeline of a patient journey from RTT. Patients who did not attend (DNA) were excluded.

## Results

Pre-intervention

Of these 143 patients, 114 (79.7%) were injected on either the right or left side (unilateral), and 28 (20.3%) required a bilateral hip injection. Out of 143, five patients did not have a breach date on the theater lists, leaving 138 patients with breach dates. Of these 138, 36 patients had been delayed beyond their RTT of 18 weeks (26.1%), implying that the target was met in 102 (71%) patients (Table 1). The mean number of days waited for injection since RTT across these 138 patients was 100 days, with a standard deviation of 42.

**Table 1:** The total number of patients and composition of those without an RTT as well as their relationship to the National Standards (with percentages in square brackets) The bolded percentage represents patients meeting the National Standard of 18 weeks. RTT: Referral to treatment.

Number of patients	143 [100%]
Without breach date	5 [3%]
RTT < 18 weeks	102 [71%]
RTT > 18 weeks	36 [26%]

Analysis of intervention

The percentage of patients treated since referral within 18 weeks was 71%, which is 24% less than the national operational standard of 95%. It is difficult to ascertain the reason for this, given that the evaluation was done retrospectively. It is likely to be a combination of patient and clinician factors. Patient factors may include booking injections via orthopedic secretaries rather than in the clinic, not attending clinic appointments, and having to rebook injection appointments. Clinician factors may include relisting and canceled lists. The study also looked at only three months’ worth of injection lists and thus may be difficult to draw conclusions on the efficacy of the services.

The absence of breach dates of five patients (3%) must also be noted. It is also difficult to ascertain the reason for this as the original electronic spreadsheets containing RTT information were not available to access. Similarly, the exact referral date was not available; fortunately, this did not affect the data collection process.

We also observed a large variation between waiting times, with a standard deviation of 42 days, or six weeks. This meant that some patients could be waiting several weeks longer than others for a hip injection intended to offer short-term relief for debilitating pain. It is difficult to ascertain why this is as this could be due to a variation in the waiting time from GP referral to be seen in secondary care as well as the time between being seen by a specialist and receiving non-admission treatment.

We did not collect information on patients who did not attend. We also did not collect information on demographics such as age and gender of patients. The reason for this was the four-week time constraint placed on the project.

**Figure 2:**
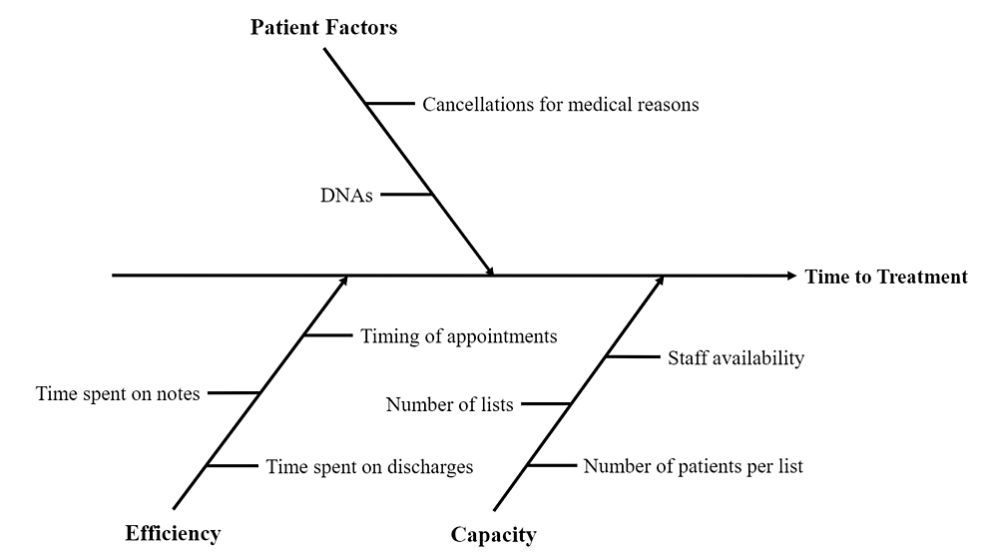
Fishbone (Ishikawa) model to determine why patients were breaching the time from referral to treatment

We adopted the fishbone (Ishikawa) model [16] to determine why patients were breaching the time from RTT (Figure 2). Categorical responses, or "tails" on the fishbone diagram, included patient factors, efficiency, and capacity. Patient factors included failing to attend appointments and cancellations for medical reasons. Efficiency included the timing of appointments as there were gaps between patients before a "wave," time spent on documentation, and the time spent on discharge summaries. Capacity included the number of available injection lists, the availability of staff for additional lists, and the number of patient slots per injection list.

**Figure 3:**
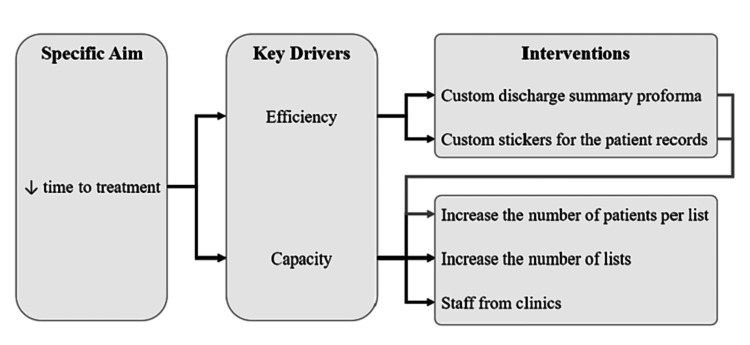
Key driver diagram to consider potential interventions to reduce the time from referral to treatment

Adopting the principle of Covey’s [17] circles of influence and concern, we sought to focus our efforts on the variables we could influence and created a key driver diagram (KDD) to consider the potential interventions to reduce the time from RTT (Figure 3). We sought to explore strategies to improve the efficiency and capacity of the injection list. Potential strategies included customizing the current discharge proformas to include the relevant documentation and information of patients, using prefilled stickers and identity stamps to reduce the time spent on documentation, and instructing patients to arrive before their allocated time to minimize gaps. Reducing the time taken to perform an IASI would also enable us to increase the capacity by increasing the number of patients per list. Additional injection lists were also considered.

**Figure 4:**
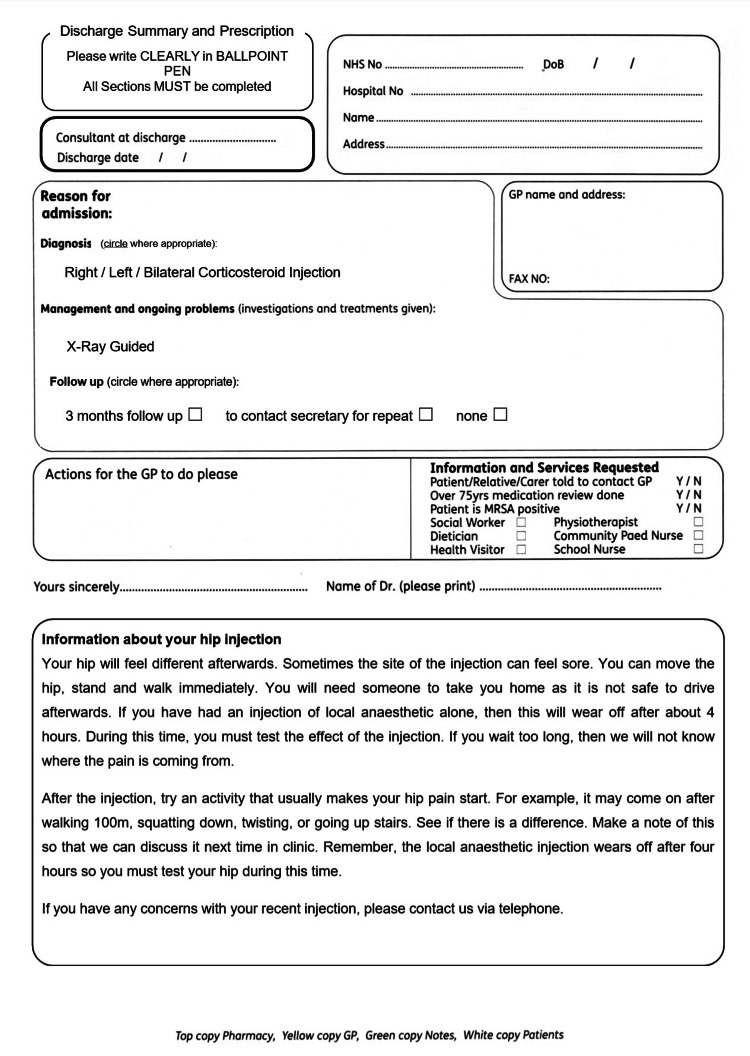
Custom discharge summary proforma

**Figure 5:**
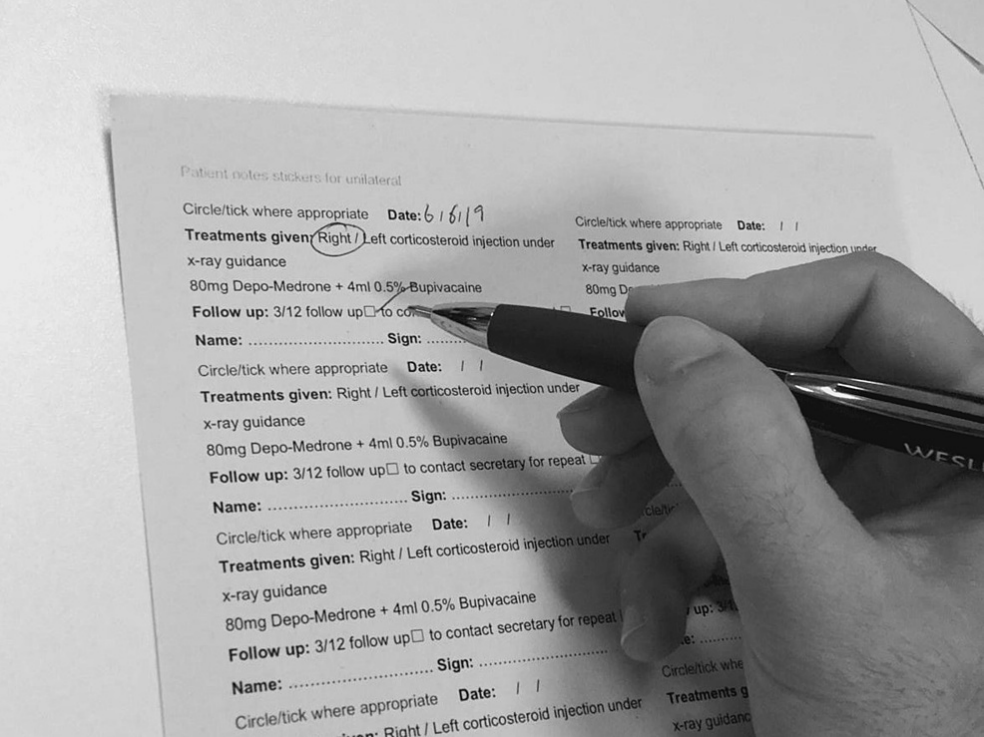
Custom stickers for the patient records

We created a custom form on the discharge proformas and included advice to patients (Figure 4). We also created a form using stickers, which could be easily transferred into the patient records (Figure 5). We trialed and amended our interventions for a few weeks and noted an anecdotal reduction in the time spent performing an IASI, which enabled us to increase the number of patients on the injection lists.

We determined the efficiency retrospectively for the pre-intervention cohort, between January and April 2019, and prospectively for the post-intervention cohort, on a single injection list. The times the patients entered and exited the fluoroscopy suite were not recorded and could not be collected retrospectively for the pre-intervention cohort. We, therefore, elected to calculate the time between the first fluoroscopy image for each consecutive patient. This was felt to provide the most comparable method, pre- and post-intervention, to determine the mean appointment length. Post-intervention data were collected once we were satisfied with the final products of the interventions.

Post-intervention

**Figure 6:**
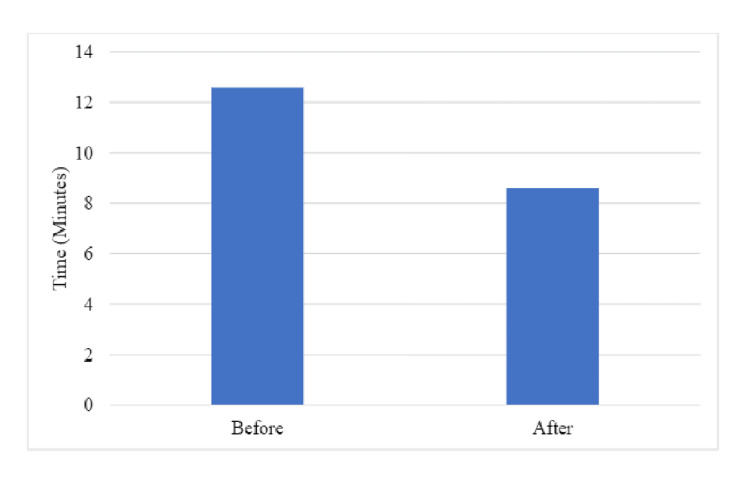
Mean time taken to perform an IASI pre- and post-intervention IASI: Intra-articular steroid injection.

Bilateral hip injections were undertaken in 28 (20%) of the 143-pre-intervention cohort and one (5%) of the 21-post-intervention cohort. The time of fluoroscopy was documented on PACS in 76 (53%) of the 143 patients in the pre-intervention cohort, with a mean (SD) interval of 12 minutes 36 seconds (7 minutes 36 seconds). Post-intervention, the mean (SD) interval was 8 minutes 36 seconds (3 minutes 12 seconds) (Figure 6). There were two 10-minute gaps waiting for the next patient to arrive in the post-intervention cohort, which was not deducted from the time of the appointments, to provide a consistent method of comparison to the pre-intervention data. The number of patients per injection list increased from a mean of 14 pre-intervention to 21 post-intervention.

## Discussion

We report a mean reduction of four minutes in the time between patients undergoing an IASI after the introduction of standardized proformas for documentation and discharge summaries. The objective of the interventions was to enhance the efficiency and hence the number of patient slots available on an injection list. As the interventions were implemented and adapted, there was an anecdotal reduction in the time taken to perform an IASI. We were, therefore, able to increase the number of patient slots available on an injection list from 14 to 21.

One-fifth of the patients in the pre-intervention cohort underwent bilateral injections, compared to only one-twentieth of those in the post-intervention group. Bilateral intra-articular injections take more time than unilateral injections, and this may account for a proportion of the reduction in the time interval. The time between fluoroscopy for each patient does not account for the gaps in waiting for the next patient to arrive. As the data collection for the pre-intervention cohort was retrospective, this information was not available; therefore, we did not deduct this from the time between fluoroscopy post-intervention. However, we noted an anecdotal increase in the time spent waiting for the next patient to arrive post-intervention, which enabled us to increase the number of patients on the injection lists. The larger standard deviation of the time in the pre-intervention cohort may be attributed to the greater number of patients (n = 143) on separate lists performed by separate clinicians, compared to a single list performed by a single clinician post-intervention (n = 21).

We determined that there were multiple factors influencing the time from RTT, using the Ishikawa model. We elected to focus on enhancing capacity through efficiency. Management simultaneously enhanced capacity through the introduction of additional injection lists. Therefore, we are unable to directly attribute any improvement in the time from RTT to our interventions. Our recommendations for future interventions would be to prospectively collect pre- and post-intervention data, including the time the patient enters and exits the radiology suite and the time spent on documentation. We acknowledge our post-intervention data is from a single injection list and, therefore, recommend that an audit should be repeated to ensure that our improvements are sustained over a three-month period.

## Conclusions

An evaluation of service was conducted in an NHS trust that offered hip IASI for its patients, and it was found that it was not meeting the national operational standard, the reason for which was not fully ascertained. Given the time constraints, strategies for improving the service were discussed, and the option to improve the documentation was explored. It was found that a streamlined prefilled pro forma, patient note labels, and stamps led to an improvement in efficiency through a reduction in the time taken to perform an IASI. This increased the list capacity through a subsequent increase in the number of patients per list. However, given the small number of patients and how it will translate into future RTT times, it is difficult to comment on the significance of this finding.

Our literature search found that while national and international guidance recommends hip IASI as short-term pharmacological pain relief, the evidence reviewed was overall poor. Hence, there is a need for further larger RCT to clearly outline their benefits. The authors recommend these simple changes in documentation to other centers to improve their IASI service. The next steps taken will be to re-audit the RTT times after using this new method of documentation for several lists and to encourage the responsible clinicians to continually evaluate this service as demand grows and new trainees perform the injection lists.
